# WHAT IS THIS COVID? COMMUNITY PERCEPTIONS OF COVID-19

**DOI:** 10.1093/geroni/igac059.1797

**Published:** 2022-12-20

**Authors:** Karen Kopera-Frye, JoJo Delgado

**Affiliations:** New Mexico State University, Las Cruces, New Mexico, United States; New Mexico State University, Las Cruces, New Mexico, United States

## Abstract

The COVID pandemic was complicated by the varied amounts and sources of information available on what exactly COVID was, e.g., CDC website, social media; some fact-based and some not. The purpose of the project was to learn how people defined COVID. The surveyed sample consisted of 155 community dwellers (M age = 45.7 years), with above average education, primarily female (66%), and slightly over one-half identifying as Caucasian and approximately 40% Latinx. The sample were all residents of the Southern Border region, having very high rates of COVID infection in this area, and a statewide mask mandate both inside and outside. The open-ended question inquiring “What is COVID?” was answered by the respondents and thematic analysis focused on two dimensions: 1) was the response factual or not (incorrect in some manner); and 2) the definition of COVID in terms of a virus, flu, etc. Interestingly, despite the median education being around 15 years, 44.5% of the sample gave an incorrect definition of COVID. The three most predominant themes emerging from the definitions were (in descending order): 1) a virus with specific facts noted; 2) a disease/infection; and 3) an affective reaction such as annoying. The results highlight the diverse conceptualizations in a very high-risk area, especially focused on oftentimes an incorrect understanding of COVID.

